# USP14 increases the sensitivity of retinoblastoma to cisplatin by mediating the ferroptosis

**DOI:** 10.1007/s00210-024-03174-9

**Published:** 2024-05-31

**Authors:** Han Liu, Qiang Gan, Yongping Lai, Zhenhui Pan, Qifang Jin, Jiayue Li, Nanye Wang, Shoufeng Jiao, Yong Chai

**Affiliations:** 1 Eye Hospital of Nanchang University, Nanchang, 330006 China; 2 Department of Ophthalmology, Jiangxi Provincial Children’s Hospital, 122 Yangming Road, Nanchang, 330006 Jiangxi Province China; 3 Pediatric Medical School, Nanchang University, Nanchang, 330031 Jiangxi China; 4 Department of Ophthalmology, The Second Affiliated Hospital of Nanchang University, Nanchang, 330006 Jiangxi China; 5 Department of Pharmacy, The First Affiliated Hospital of Nanchang University, No.17, Yongwai Road, Nanchang, 330006 China

**Keywords:** Retinoblastoma, USP14, Ferroptosis, FASN, Drug resistance

## Abstract

The aim of this study is to explore the function of USP14 on the sensitivity of retinoblastoma (RB) to cisplatin (DDP) and the underlying mechanism. USP14 was knockdown in Y79 cells by transfecting three siRNAs (si-USP14-1, si-USP14-2, and si-USP14-3), with si-USP14 NC as the negative control. si-USP14-3 was selected by results of Western blotting. The CCK-8 assay was used to detect the IC50 of Y79 cells and the growth curve. The cell cycle, cell apoptosis, and ROS level were measured by flow cytometry. The expression level of P-GP, ERCC1, survivin, GPX4, FTH1, ACSL4, NOX1, COX2, and FASN was determined by the Western blotting assay. CO-IP assay was utilized to evaluate the interaction between USP14 and FASN. The IC50 of DDP in Y79 cells and Y79/DDP cells was 7.83 µM and 24.67 µM, respectively. Compared to control and si-USP14 NC groups, increased apoptotic rate and ROS level, and arrested cell cycle in S phase were observed in USP14-knockdown Y79 cells. Compared to control and si-USP14 NC groups, increased apoptotic rate and arrested cell cycle in G0/G1 phase were observed in USP14-knockdown Y79/DDP cells. Compared to control, increased ROS level was observed in USP14-knockdown Y79/DDP cells. Compared to the si-USP14 NC groups, extremely downregulated P-GP, ERCC1, survivin, GPX4, FTH1, NOX1, COX2, and FASN were observed in USP14-knockdown Y79 cells or Y79/DDP cells, accompanied by the elevated expression of ACSL4. The interaction between USP14 and FASN was identified according to the result of CO-IP assay. By silencing USP14 in Y79 and Y79/DDP cells, levels of resistance-related proteins (P-GP, ERCC1, and survivin), ferroptosis-related proteins (FTH1 and GPX4), and lipid metabolism-related proteins (NOX1, COX2, and FASN) were dramatically reduced, accompanied by enhanced ROS level, increased apoptosis, and restrained DNA content, indicating that USP14 might suppress the DDP resistance in RB by mediating ferroptosis, which is an important target for treating RB.

## Introduction

Retinoblastoma (RB) is an aggressive and most common intraocular cancer in children, mainly caused by mutations in the tumor suppressor gene RB transcriptional corepressor 1 (RB1) (Russo et al. 2022). RB is regularly observed before the age of 5 and is most commonly seen in children under the age of 2 (Roy 2021). RB accounts for 3% of childhood cancers, and the global survival rate of RB patients is less than 30% (Dean et al. 2014). Although treatment options and recommendations depend on the type and stage of RB, surgical enucleation and postoperative platinum-based chemotherapy, including systemic, subconjunctival, intra-arterial, and intravitreal routes, are still the main treatment options currently (Ancona-Lezama et al. 2020). Nevertheless, due to the early metastasis of RB, most children are not sensitive to chemotherapy, resulting in poor prognosis and the low long-term survival rate. Therefore, it is particularly important to explore the mechanism of RB resistance to chemotherapy drugs. Cisplatin (DDP) is a basic chemotherapy drug for clinical treatment of malignant tumors, which shows promising effects on a variety of tumors. It is clinically used in breast cancer (Mendoza and Grossniklaus 2015), non-small cell cancer (Pritchard et al. 2016), and gastric cancer (Shields et al. 2014), etc. However, the secondary drug resistance of tumor cells to DDP seriously affects the clinical efficacy and prognosis in patients. Therefore, it is urgent to identify novel targets and explore the mechanisms of drug resistance of DDP in RB to improve the therapeutic effect and overcome the resistance to RB therapy.

Ubiquitination is one of protein post-translational modifications that control intracellular protein degradation through a series of procedures, including substrate recognition, ubiquitin binding, and proteasomal degradation of ubiquitinated substrates. USP is the largest family of deubiquitinating enzymes (DUBs), which are involved in a variety of cellular processes, including cell cycle, cell proliferation, cell differentiation, transcriptional regulation, and regulation of plasma membrane receptors. USP14 is one of the three deubiquitinating enzymes (USP14, Uch37, and Rpn11) that bind to the proteasome and can be recruited to the 19S particle to avoid its recognition and degradation by the proteasome via cutting the ubiquitin chain on the protein substrate (de Poot et al. 2017; Lee et al. 2011). Correspondingly, inhibition of USP14 is reported to improve proteasomal degradation activity (Lee et al. 2010). Studies have shown that USP14 is overexpressed in a variety of cancers and neurodegenerative diseases, which plays an important regulatory role in cell activities such as cell proliferation and apoptosis, DNA damage repair, and signaling transduction. However, the specific molecular mechanisms have not been elucidated (Boselli et al. 2017; Liu et al. 2017). Despite the importance of USP14 in cell physiology and disease, few studies reported the role of USP14 in energy metabolism, which need to be further studied. A variety of death modes are reported on malignant tumors, among which ferroptosis is a newly discovered iron-dependent cell death mode by accumulation of lipid peroxides, which is closely related to the occurrence, development, and drug resistance of malignant tumors. However, the regulatory effect of USP14 on ferroptosis in DDP-resistant tumor cells has not been studied.

The present study aim is to explore the effects of USP14 knockdown on oxidative stress, ferroptosis, and drug resistance in human RB cell line Y79 cells and DDP-resistant Y79 (Y79/DDP) cells.

## Materials and methods

### Cells and treatments

Y79 cells and Y79/DDP cells were purchased from ATCC (USA) and cultured in DMEM medium supplemented with 10% FBS and 1% penicillin-streptomycin under the condition of 37℃ and 5% CO2. To explore the function of USP14 in RB cells, a siRNA targeting USP14 (si-USP14) was designed and transfected into Y79 cells to establish the USP14-knockdown Y79 cells, taking the si-USP14 NC as the negative control. Three si-USP14s were designed for the screening of optimized USP14-knockdown Y79 cells. For the screening of siRNA, 5 groups were divided: Control, Si-USP14 NC, Si-USP14-1, Si-USP14-2, and Si-USP14-3. In the control group, Y79 or Y79/DDP cells were cultured with blank medium. In the Si-USP14 NC, Si-USP14-1, Si-USP14-2, and Si-USP14-3 groups, Y79 or Y79/DDP cells were transfected with Si-USP14 NC, Si-USP14-1, Si-USP14-2, and Si-USP14-3, respectively. To evaluate the role of USP14 in DDP resistance, 3 groups were divided: Control, Si-USP14 NC, and Si-USP14. In the control group, Y79 or Y79/DDP cells were cultured with blank medium. In the Si-USP14 NC and Si-USP14 groups, Y79 or Y79/DDP cells were transfected with Si-USP14 NC and Si-USP14-3, respectively.

### CCK-8 assay

The cell viability was evaluated using the CCK-8 commercial kit (KGA317, KeyGEN, China). Cells were seeded into individual wells of a 96-well plate and allowed to adhere for a period of 24 h. Subsequently, 10 µl of CCK8 solution was added to each well. Following a 2-h incubation period, the optical density (OD) value of each well was measured using a microplate reader (WD-2012B, LIUYI, China).

### The detection of cell cycle using the flow cytometry

The Cell Cycle Staining Kit (CCS102, MULTI SCIENCES, China) was utilized to detect the cell cycle. 1 × 10^6^ cells were collected and washed by PBS buffer, which were then added with cell cycle reagent to be incubated in the dark for 30 min. Then, cells were loaded onto the flow cytometry (NovoCyte 2060R, ACEA Biosciences, China) for the analysis of cell cycle.

### The detection of apoptosis using the flow cytometry

The Annexin V-FITC/PI Apoptosis Kit (AP101-100-kit, MULTI SCIENCES, China) was utilized to evaluate the apoptosis. 1 × 10^6^ cells were collected and washed by PBS buffer, which were then resuspended with 300 µl pre-cold 1× Annexin V-FITC binding buffer. Then, cells were introduced with 5 µl Annexin V-FITC reagent and 10 µl PI reagent, followed by 10-min incubation in the dark at room temperature. Lastly, cells were loaded onto the flow cytometry (NovoCyte 2060R, ACEA Biosciences, China) for the analysis of apoptosis.

### The detection of ROS level using the flow cytometry

The Reactive Oxygen Species Assay Kit (KGT010-1100assays, MULTI SCIENCES, China) was used to determine the ROS level. Cells were collected and added with 10 µM DCFH-DA reagent, followed by incubation at 37℃ for 20 min. After 3 times of washing, cells were resuspended with 300 µl PBS, followed by loading onto the flow cytometry (NovoCyte 2060R, ACEA Biosciences, China) for the analysis of ROS level.

### Western blotting assay

The BCA kit (Cwbio, Jiangsu, China) was utilized to quantify the protein isolated from cells and tissues, followed by being separated with the 12% SDS-PAGE. The separated protein was transferred from the gel to the PVDF membrane, which was further introduced with 5% skim milk. Then, the membrane was introduced with the primary antibody against P-GP (1:1000, 22336-1-AP, Proteintech, USA), ERCC1 (1:1000, DF7255, Affinity, USA), NOX1 (1:1000, 17772-1-AP, Proteintech, USA), COX2 (1:1000, bs-10411R, Bioss, USA), GPX4 (1:1000, 67763-1-Ig, Proteintech, USA), FTH1 (1:1000, DF6278, Affinity, USA), ACSL4 (1:1000, DF12141, Affinity, USA), FASN (1:1000, 66591-1-Ig, Proteintech, USA), USP14 (1:1000, sc-515,812, Santa Cruz, USA), and β-actin (1:1000, HC201, TransGen Biotech, China). The second antibody (1:2000, GB23303, Servicebio, China) was subsequently added to be incubated for 90 min. Finally, ECL reagent was added to expose the bands, which were further quantified with the ImageJ software.

### Coimmunoprecipitation (CO-IP) assay

A CO-IP commercial kit (C600689, Sangon Biotech, China) was utilized to evaluate the binding between USP14 and FASN. Cells were lysed with cell lysate, and part of the lysate was used as input samples. A 700 μl lysate was incubated with USP14 antibody for 4 h at 4 ℃, with IgG protein as a negative control. The finished incubation liquid was transferred to A Protein A/G agarose (Thermo Fisher, USA)-treated purification column (Thermo Fisher, USA), followed by incubated overnight at 4 ℃ and centrifuged at 12,000 rpm for 30 s at 4 ℃ to remove the supernatant. The agarose beads were washed with IP buffer (Thermo Fisher, USA), centrifuged at 12,000 rpm for 30s, and added with 50 μl loading buffer, followed by centrifugation at 12,000 rpm for 30 s in a water bath at 95℃ for 5 min. The centrifugation products were collected, and the protein expression level of FASN was detected by the Western blotting assay.

### Statistical analysis

Mean ± SD was utilized to present data, which was analyzed using the one-way ANOVA method with the software of GraphPad Prism 8.0.1 software. *P* < 0.05 was considered to be a statistically significant difference.

## Results

### The identification of USP14-knockdown efficacy

**Fig. 1:**
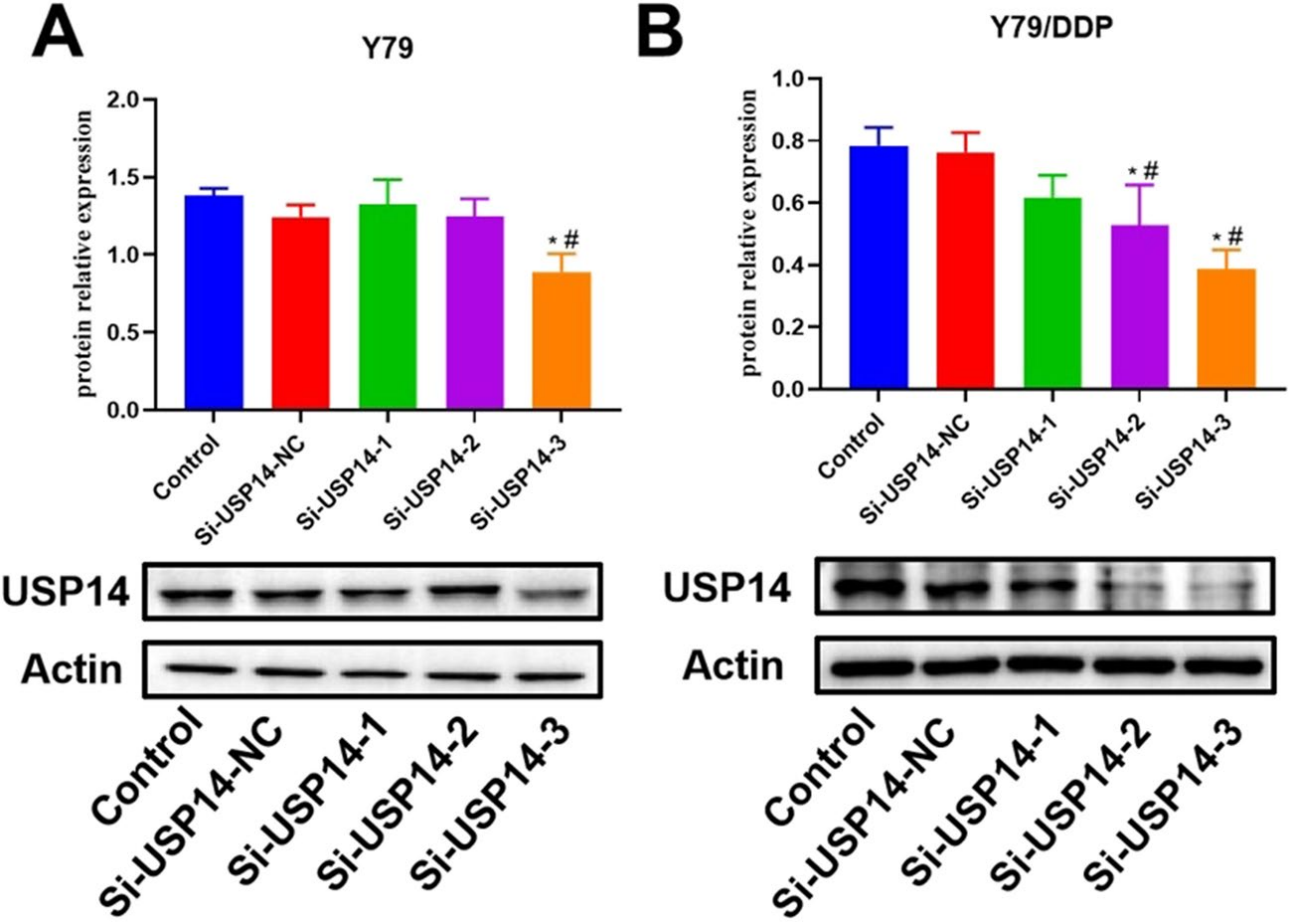
The expression level of USP14 in Y79 (**A**) and Y79/DDP cells (**B**) was detected by the Western blotting assay (*P < 0.05 vs. Control, ^#^*P* < 0.05 vs. Si-USP14-NC)

As shown in Fig. 1, in Y79 cells, compared to control and si-USP14 NC groups, USP14 was markedly downregulated in the Si-USP14-3 group. Furthermore, in Y79/DDP cells, compared to control and si-USP14 NC groups, USP14 was markedly downregulated in the Si-USP14-2 and Si-USP14-3 groups. Therefore, Si-USP14-3 was picked in the subsequent experiments.

### IC50 of Y79 cells and Y79/DDP cells to DDP

**Fig. 2:**
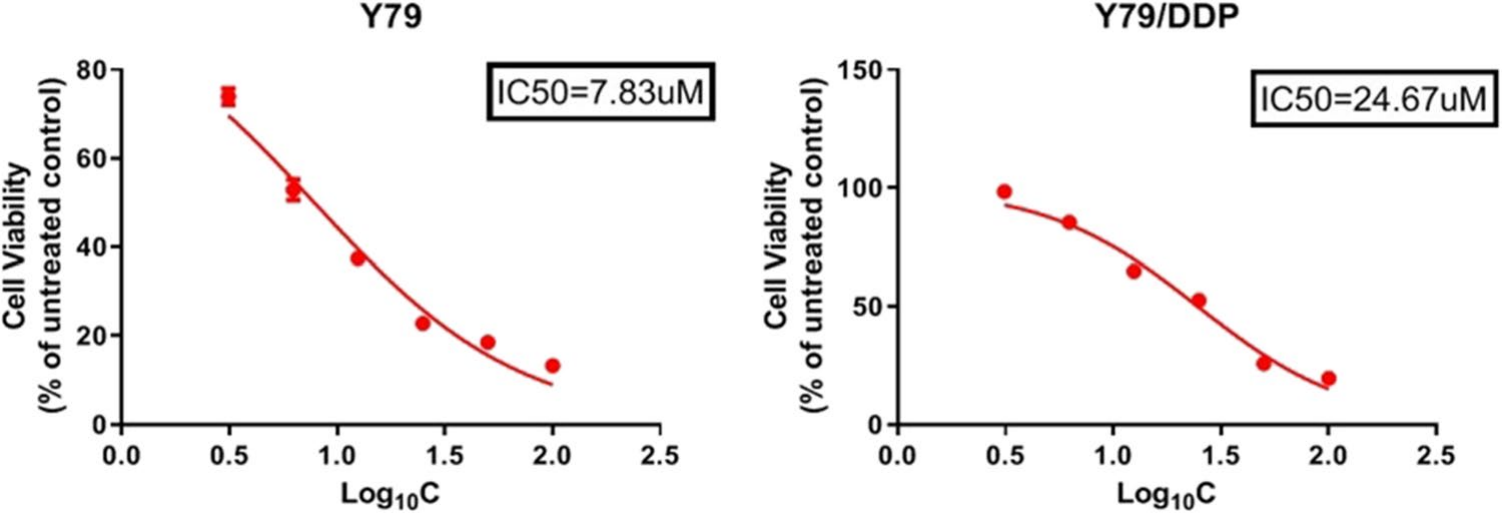
The IC50 of DDP on Y79 and Y79/DDP cells was measured by the CCK-8 assay

As shown in Fig. 2, the IC50 of DDP on Y79 cells and Y79/DDP cells was 7.83 µM and 24.67 µM, respectively, suggesting the successfully establishment of Y79/DDP cells.

### The impact of USP14 on the cell cycle in Y79 cells and Y79/DDP cells

**Fig. 3:**
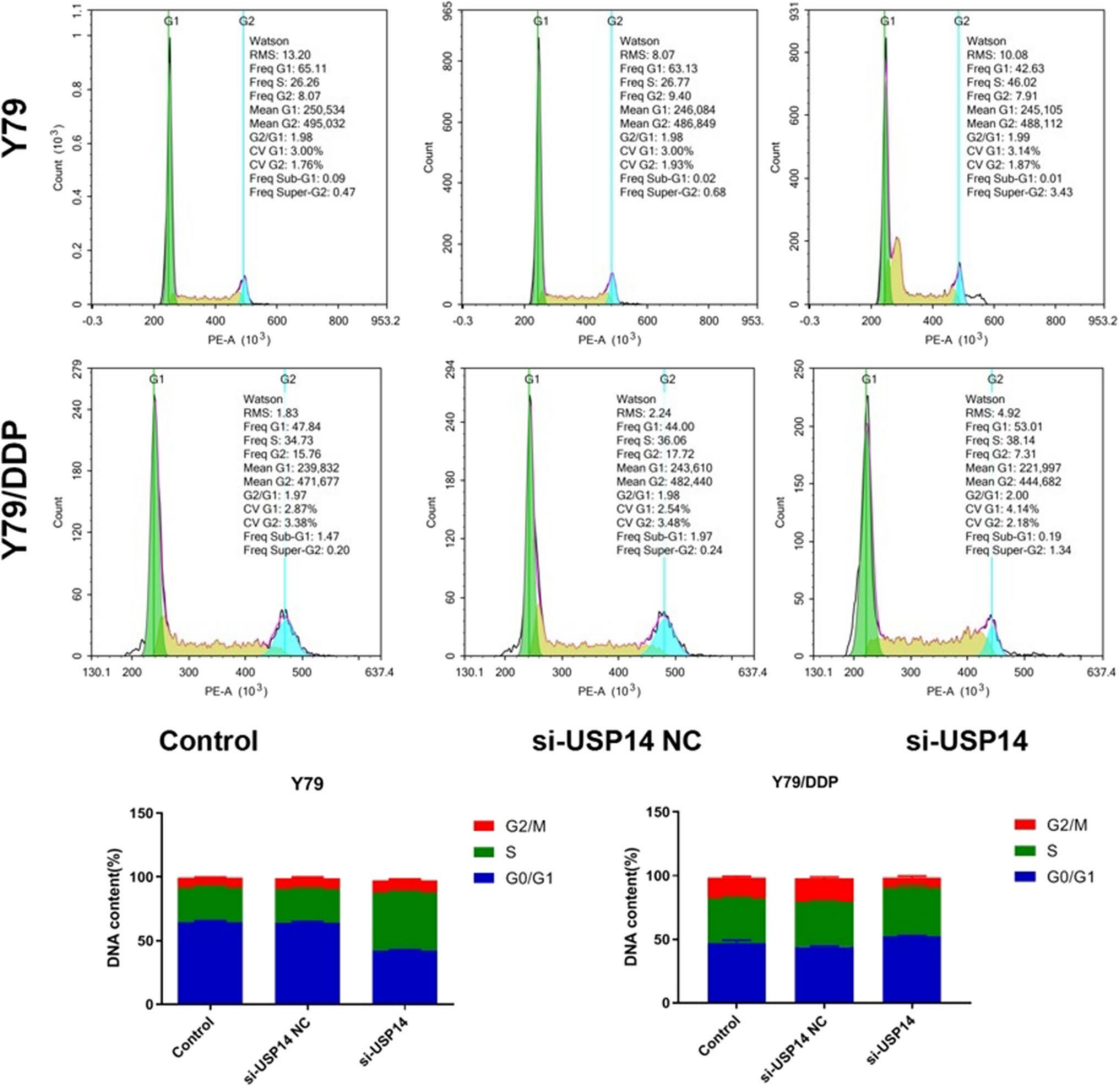
The cell cycle of Y79 and Y79/DDP cells was evaluated by the flow cytometry

To evaluate the role of USP14 in DDP resistance, 3 groups were divided: Control, Si-USP14 NC, and Si-USP14. In the control group, Y79 or Y79/DDP cells were cultured with blank medium. In the Si-USP14 NC and Si-USP14 groups, Y79 or Y79/DDP cells were transfected with Si-USP14 NC and Si-USP14-3, respectively. Flow cytometry was utilized to evaluate the cell cycle in each group. As shown in Fig. 3, in Y79 cells, compared to control and si-USP14 NC groups, cells were arrested at the S phase by the knockdown of USP14. Furthermore, in Y79/DDP cells compared to control and si-USP14 NC groups, cells were arrested at the G0/G1 phase by the knockdown of USP14. More Y79 cells were arrested at the phase of DNA synthesis by knocking down USP14, while more Y79/DDP cells were arrested at the phase of DNA replication and cell division by knocking down USP14.

### The impact of USP14 on the apoptosis in Y79 cells and Y79/DDP cells

**Fig. 4:**
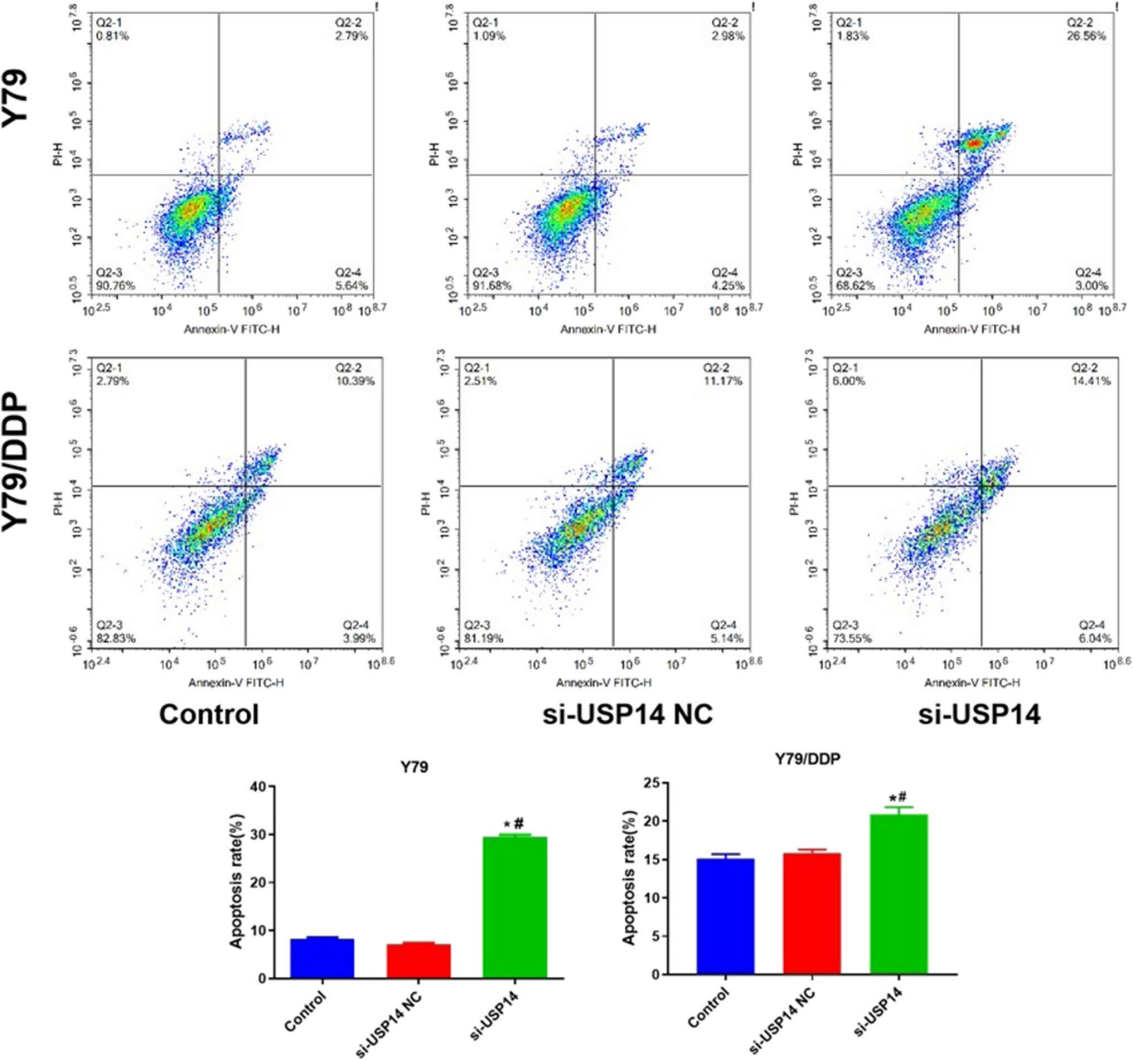
The apoptosis in Y79 and Y79/DDP cells was evaluated by the flow cytometry (*P < 0.05 vs. Control, ^#^*P* < 0.05 vs. Si-USP14-NC)

As shown in Fig. 4, in both Y79 cells and Y79/DDP cells, compared to control and si-USP14 NC groups, the apoptotic rate was extremely increased by the knockdown of USP14. Comparing to Y79 cells, the increase fold of apoptosis rate in Y79/DDP cells was smaller following silencing USP14, suggesting a more significant influence of USP14 on the apoptosis in Y79 cells than in Y79/DDP cells.

### The impact of USP14 on the ROS production in Y79 cells and Y79/DDP cells

**Fig. 5:**
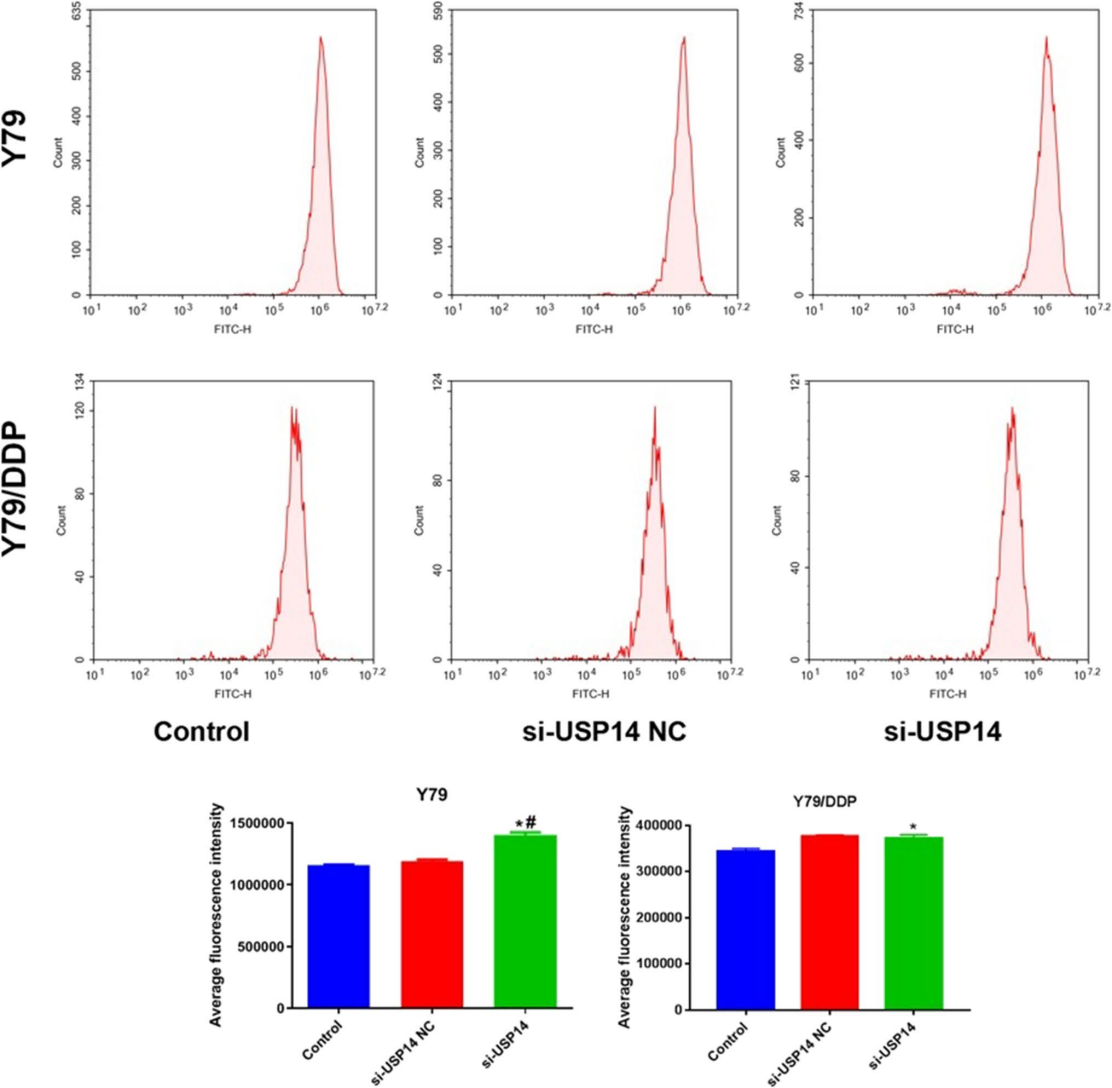
The ROS level in Y79 and Y79/DDP cells was detected by the flow cytometry (*P < 0.05 vs. Control, ^#^*P* < 0.05 vs. Si-USP14-NC)

As shown in Fig. 5, in both Y79 cells and Y79/DDP cells, compared to control and si-USP14 NC groups, the release of ROS was markedly elevated by the knockdown of USP14. Similarly, comparing to Y79 cells, the increase fold of ROS level in Y79/DDP cells was smaller following silencing USP14, suggesting a more significant influence of USP14 on the ROS production in Y79 cells than in Y79/DDP cells.

### The impact of USP14 on the resistance-, ferroptosis-, and lipid metabolism-related proteins in Y79 cells and Y79/DDP cells

**Fig. 6:**
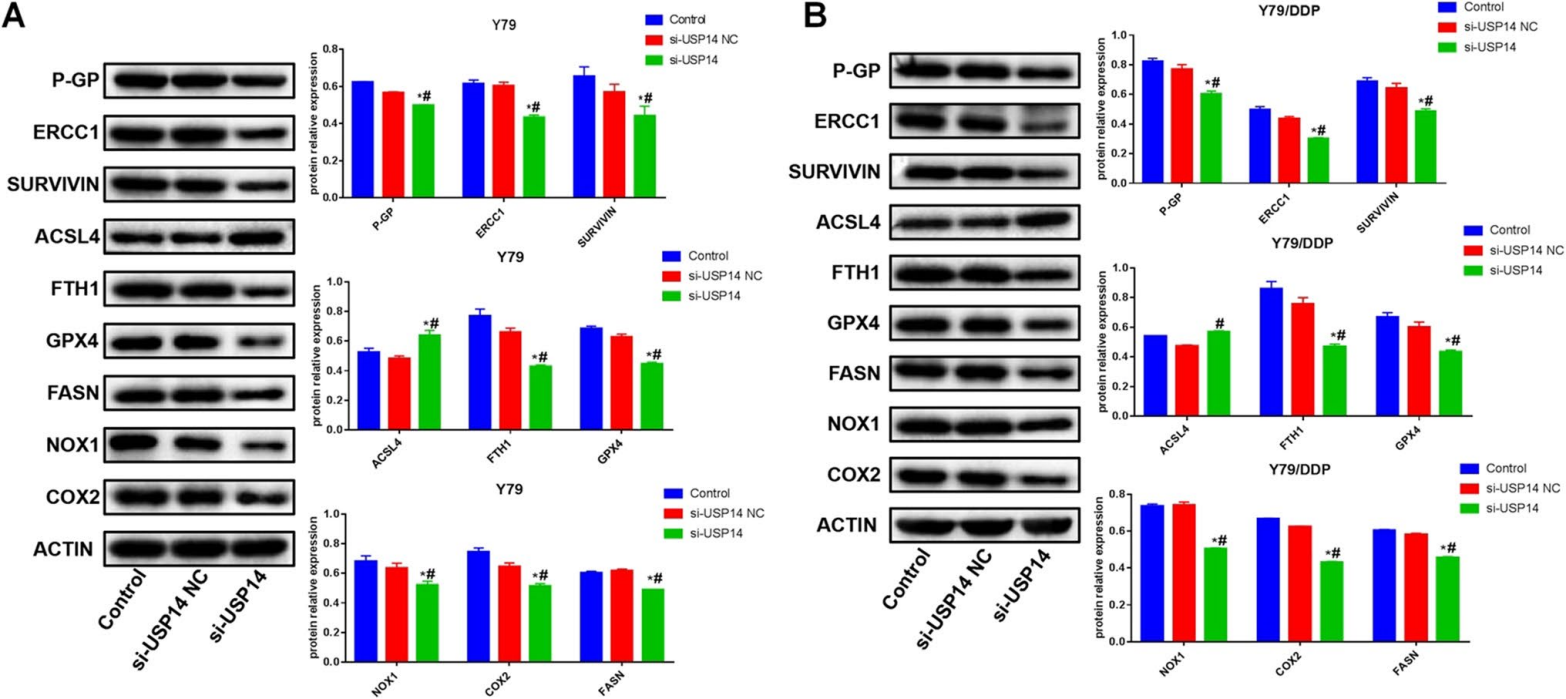
The expression level of P-GP, ERCC1, survivin, GPX4, FTH1, ACSL4, NOX1, COX2, and FASN in Y79 (**A**) and Y79/DDP (**B**) cells was determined by the Western blotting assay (*P < 0.05 vs. Control, ^#^*P* < 0.05 vs. Si-USP14-NC)

As shown in Fig. 6, in both Y79 cells and Y79/DDP cells, compared to control and si-USP14 NC groups, P-GP, ERCC1, surviving, FTH1, GPX4, FASN, NOX1, and COX2 were found downregulated, while ACSL4 was upregulated by the knockdown of USP14.

### The interaction between USP14 and FASN was identified by the CO-IP assay

**Fig. 7:**
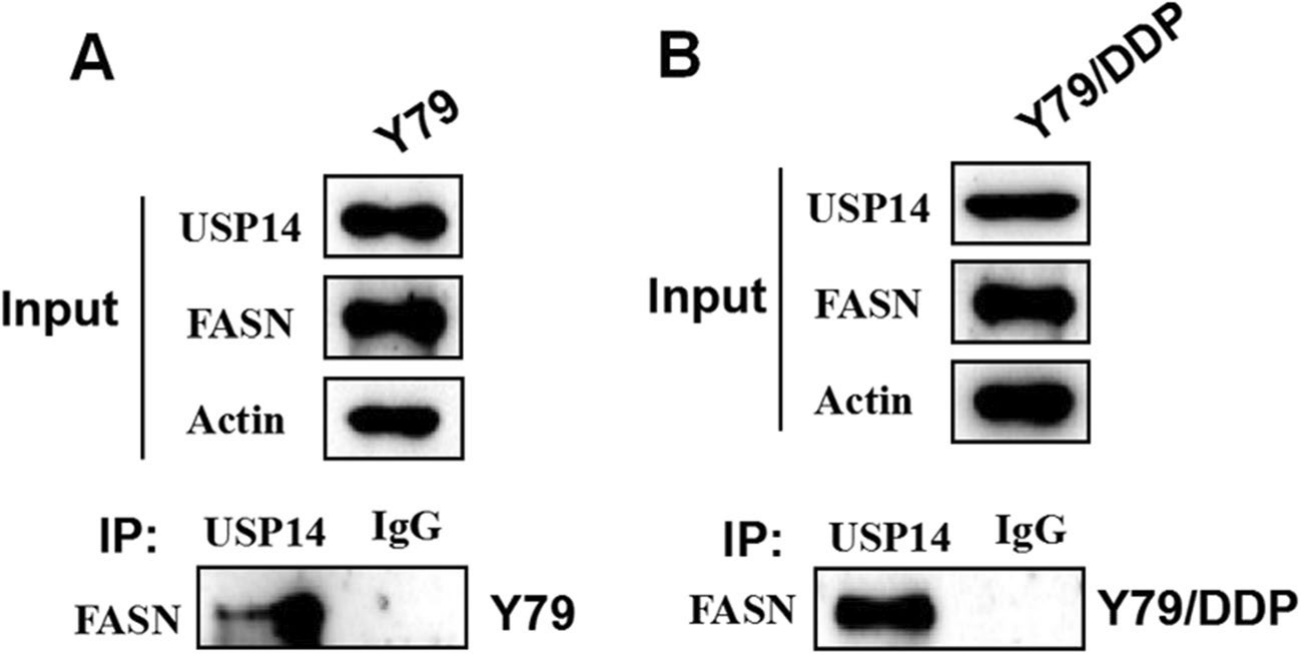
The interaction between USP14 and FASN in Y79 and Y79/DDP cells was determined by the CO-IP assay

As shown in Fig. 7, in both Y79 cells and Y79/DDP cells, USP14 was observed in pull-down proteins. Furthermore, FASN was detected in pull-down proteins using the FASN antibody, while FASN was not observed in the IgG group, suggesting that an interaction was identified between USP14 and FASN in Y79 cells and Y79/DDP cells.

## Discussion

Discussing cancer diagnosis and treatment is a challenging task and treatments for rare cancers are particularly challenging for patients, caregivers, and even clinicians. The development of chemoresistance, including primary, adaptive, and acquired resistance, remains a serious obstacle to the treatment of RB patients (Ancona-Lezama et al. 2020).

Lee et al. claim that USP14 plays an important role in the cell cycle by maintaining ubiquitin (Ub) homeostasis. In mouse embryonic fibroblasts with permanent deletion of USP14, Ub homeostasis is significantly disturbed and proteasome activity is enhanced, which leads to abnormal degradation of Cyclin A and B1, resulting in prolonged interphase and delayed proliferation. Similarly, the knockdown of USP14 in Drosophila results in delayed eye development and reduced mitotic activity (Lee et al. 2018). In autophagy-deficient prostate cancer cells, USP14 acts as a regulator of DNA damage response by negatively regulating RNF168-dependent ubiquitination signaling and disrupting DNA damage response signaling in autophagy-deficient cells (Sharma et al. 2018). In the present study, we found that DNA arrested at G0/G1 phase was observed in Y79 cells after the downregulation of USP14, while DNA was arrested in G2/M phase in Y79/DDP cells following the knockdown of USP14. Numerous studies have shown that USP14 is amplified and overexpressed in various types of malignant tumors. The abnormal activity of USP14 is closely related to the initiation, development, and drug resistance of cancer. Overexpression of USP14 promoted cell proliferation and migration, while downregulation of USP14 inhibited tumor cell proliferation, migration and invasion, with declined cell apoptosis. USP14 plays an oncogenic role in various types of cancer, and its inhibitors have shown significant effects in a variety of anticancer studies. The expression of USP14 is negatively correlated with prognosis, which plays a crucial role in cancer development and has been recognized as a new therapeutic target for cancer (Zhu et al. 2016). In this study, we found that after downregulation of USP14, the apoptotic rate was significantly increased in Y79 and Y79/DDP cells.

P-glycoprotein (P-GP) is a membrane channel that pumps out antinuclear drugs, thereby preventing drug-induced toxicity. P-GP is the encoded product of multidrug resistance gene MDR-1, which actively transports drugs out of the cell through ATP hydrolysis, eventually leading to tumor drug resistance (Chufan et al. 2016; Chen et al. 2016). Survivin is the strongest inhibitor of apoptosis discovered currently. It is a member of the inhibitor of apoptosis protein family and plays an important role in tumor cell proliferation, apoptosis, and angiogenesis (Yang Jiaojiao 2017). Wall et al. (2003) confirmed that the phosphorylation of Survivin on the site of Thr34 was essential for maintaining cell division and cell activity. After introducing artificial Survivin with T34 mutant into cervical cancer cells, the apoptosis is facilitated and the sensitivity of tumor cells to chemotherapy is increased. Excision repair cross-complementing gene 1 (ERCC1) plays an important role in the DNA repair pathway, which recognizes and repairs damaged gene fragments. The expression level of ERCC1 is considered a biomarker of the entire DNA nucleic acid excision repair activity. The high expression of ERCC1 protein may be the main mechanism of drug resistance to platinum-based chemotherapy in tumor patients (Huang Yuliang 2019). Ozcan et al. (2013) confirmed that in patients with bladder cancer who received platinum-based NAC, high ERCC1 expression was considered to be an indicator of poor prognosis of bladder cancer. In the present study, we found that the protein expression of P-GP, ERCC1, and survivin was significantly decreased in Y79 and Y79/DDP cells after the downregulation of USP14.

Ferroptosis is a type of programmed cell death, which is characterized by iron ion dependence and increased lipid peroxidation. Ferroptosis is reported to be involved in a variety of diseases, such as malignant tumors, ischemia-reperfusion injury, and neurodegeneration. It is believed that GSH is the main substrate of glutathione peroxidase 4 (GPX4), and GPX4 is the key regulator of ferroptosis. Decreased activity of GPX4 can be induced by GSH consumption, which is not sufficient for the elimination of harmful lipid peroxides. Acyl-CoA synthetase long-chain family member 4 (ACSL4) is considered to be an important contributor to ferroptosis (Luo Manhui and Guo 2021) and plays an important role in the biosynthesis and remodeling of phosphatidylethanolamine, which affects the transmembrane transport of polyunsaturated fatty acids (Wu et al. 2014). Li et al. found that inhibition of ACSL4 in advance effectively suppressed ferroptosis and cell damage during intestinal ischemia-reperfusion (Romani et al. 2021). Ferritin heavy chain 1 (FTH1) is another a recognized ferroptosis marker molecule, which participates in iron phagocytosis to be degraded. The decreased iron storage ability of FTH1 will result in the accumulation of iron ions in the body, and produce excessive oxygen free radicals through Fenton reaction, leading to ferroptosis. In the present study, we found that the protein expression of FTH1 and GPX4 was significantly decreased and the protein expression of ACSL4 was significantly increased in Y79 and Y79/DDP cells after the knockdown of USP14. Large accumulation of lipid ROS is another inducer that directly triggers ferroptosis, a process that can be prevented by lipophilic antioxidants and iron chelators. NOXs provide a large amount of ROS in erastin-induced ferroptosis. Studies have found that PTGS2, the gene encoding cyclooxygenase-2 (COX-2), is a unique and widely used marker for erastin- or RSL3-induced ferroptosis in tumors (Yang et al. 2014). In the present study, we found that after the downregulation of USP14, the protein expression of NOX 1 and COX-2 was significantly decreased, and the ROS production was significantly increased in Y79 and Y79/DDP cells. However, in Y79/DDP cells, there is no significant difference regarding ROS level between the si-USP14 NC and si-USP14 groups. We suspected that compared to control, the si-USP14 NC was an external substance to Y79/DDP cells, which might induce an increase in the ROS production due to stress response. The assay will be repeated to confirm our hypothesis.

Studies have shown that fatty acids (FAs) are essential components of all biofilm lipids and important substrates for energy metabolism. FAs are metabolized by animals from two sources: exogenous (dietary) and endogenous FAs. Biosynthesis of endogenous FAs is catalyzed by a 250–270 kD multifunctional homodimeric fatty acid synthase (FASN). Tumor cells metabolize glucose at a rate that exceeds bioenergy requirements by switching from oxidative to glycolytic metabolism. In this case, tumor cells redirect excess glycolytic end product pyruvate to de novo FA synthesis. There are differences in the results of lipid synthesis by FASN between normal and cancer tissues. FASN in normal cells is responsible for the synthesis of lipids in the form of triacylglycerol, which are primarily used for energy storage. However, in tumor cells, lipids de novo synthesized by FASN are preferentially chosen as phospholipids that may be involved in cell signaling. Proteomics results showed that FASN was overexpressed in breast cancer cell lines with a multidrug resistant phenotype, the expression of which increased with the level of drug resistance. Furthermore, FASN activity was found to be upregulated in gemcitabine-selected pancreatic cancer cell line G3K cells, although with no significant changes on the expression level of FASN. Moreover, upregulation of FASN in pancreatic cancer cells is found to facilitate gemcitabine resistance, supporting a possible role of FASN in acquired gemcitabine resistance. The finding of increased FASN activity in gemcitabine-selected cell lines and its role in gemcitabine resistance further confirms the role of FASN in acquired resistance and suggests that FASN activity may be post-translational regulated (Mendoza and Grossniklaus 2015).

Subsequently, we explored the specific molecular mechanism of USP14 regulating FASN. It is reported that ferroptosis is closely related to USP14 and lipid metabolism (Shields et al. 2014; de Poot et al. 2017). Theoretically, the abundance and location of FASN-mediated polyunsaturated fatty acids determine the degree of lipid peroxidation in cells, and thus the degree of ferroptosis (de Poot et al. 2017). In addition, knockdown of FASN shows a protective effect on ferroptosis in HT22 cells. However, HT22 cells are not tumor cells and the specific mechanism may be different from that in tumor cells (Lee et al. 2011). Multiple evidences have shown that FASN is closely related to ferroptosis of cells. It has been reported that interference of related genes in drug-resistant cells promotes drug-induced ferroptosis to achieve antitumor effects. Therefore, ferroptosis mediated by FASN might be a potential drug resistance target (Lee et al. 2010). In the present study, we found that the protein expression of FASN was significantly decreased in Y79 and Y79/DDP cells after USP14 knockdown, and the interaction between FASN and USP14 was confirmed by the CO-IP assay. In our future work, the specific agonist of USP14 will be designed to evaluate the potential anti-RB property using the in vitro and in vivo assays.

In summary, USP14 increased the sensitivity of RB to DDP by mediating the ferroptosis, which might be an important target for the treatment of DDP resistance in RB.

## Data Availability

No datasets were generated or analysed during the current study.

## References

[CR1] Ancona-Lezama D, Dalvin LA, Shields CL (2020) Modern treatment of retinoblastoma: a 2020 review. Indian J Ophthalmol 68(11):2356–2365 33120616 10.4103/ijo.IJO_721_20 PMC7774148

[CR2] Boselli M, Lee BH, Robert J, Prado MA, Min SW, Cheng C, Silva MC, Seong C, Elsasser S, Hatle KM, Gahman TC, Gygi SP, Haggarty SJ, Gan L, King RW, Finley D (2017) An inhibitor of the proteasomal deubiquitinating enzyme USP14 induces tau elimination in cultured neurons. J Biol Chem 292(47):19209–19225 28972160 10.1074/jbc.M117.815126 PMC5702663

[CR3] Chen Z, Shi T, Zhang L, Zhu P, Deng M, Huang C, Hu T, Jiang L, Li J (2016) Mammalian drug efflux transporters of the ATP binding cassette (ABC) family in multidrug resistance: a review of the past decade. Cancer Lett 370(1):153–164 26499806 10.1016/j.canlet.2015.10.010

[CR4] Chufan EE, Kapoor K, Ambudkar SV (2016) Drug-protein hydrogen bonds govern the inhibition of the ATP hydrolysis of the multidrug transporter P-glycoprotein. Biochem Pharmacol 101:40–53 26686578 10.1016/j.bcp.2015.12.007 PMC4753104

[CR5] de Poot SAH, Tian G, Finley D (2017) Meddling with fate: the proteasomal deubiquitinating enzymes. J Mol Biol 429(22):3525–3545 28988953 10.1016/j.jmb.2017.09.015 PMC5675770

[CR6] Dean M, Bendfeldt G, Lou H, Giron V, Garrido C, Valverde P, Barnoya M, Castellanos M, Jimenez-Morales S, Luna-Fineman S (2014) Increased incidence and disparity of diagnosis of retinoblastoma patients in Guatemala. Cancer Lett 351(1):59–63 24814393 10.1016/j.canlet.2014.04.023 PMC4490907

[CR7] Huang Yuliang ZB (2019) Research progress on the relationship between excision repair cross-complementing gene 1 and tumors. Oncol Progress 17(4):376–379

[CR8] Lee BH, Lee MJ, Park S, Oh DC, Elsasser S, Chen PC, Gartner C, Dimova N, Hanna J, Gygi SP, Wilson SM, King RW, Finley D (2010) Enhancement of proteasome activity by a small-molecule inhibitor of USP14. Nature 467(7312):179–184 20829789 10.1038/nature09299 PMC2939003

[CR9] Lee JH, Park S, Yun Y, Choi WH, Kang MJ, Lee MJ (2018) Inactivation of USP14 perturbs ubiquitin homeostasis and delays the cell cycle in mouse embryonic fibroblasts and in fruit fly drosophila. Cell Physiol Biochem 47(1):67–82 29763934 10.1159/000489750

[CR10] Lee MJ, Lee BH, Hanna J, King RW (2011) Trimming of ubiquitin chains by proteasome-associated deubiquitinating enzymes. Mol Cell Proteomics 10(5):R110 003871 20823120 10.1074/mcp.R110.003871 PMC3098602

[CR11] Liu N, Kong T, Chen X, Hu H, Gu H, Liu S, Chen X, Yang Q, Li A, Xiong X, Zhang Z (2017) Ubiquitin-specific protease 14 regulates LPS-induced inflammation by increasing ERK1/2 phosphorylation and NF-kappaB activation. Mol Cell Biochem 431(1–2):87–96 28364380 10.1007/s11010-017-2978-0

[CR12] Luo Manhui PH, Guo Deyin (2021) Mechanisms of ferroptosis and its application in cancer therapy. Life Chem 41(01):10–18

[CR13] Mendoza PR, Grossniklaus HE (2015) The biology of retinoblastoma. Prog Mol Biol Transl Sci 134:503–516 26310174 10.1016/bs.pmbts.2015.06.012

[CR14] Ozcan MF, Dizdar O, Dincer N, Balci S, Guler G, Gok B, Pektas G, Seker MM, Aksoy S, Arslan C, Yalcin S, Balbay MD (2013) Low ERCC1 expression is associated with prolonged survival in patients with bladder cancer receiving platinum-based neoadjuvant chemotherapy. Urol Oncol 31(8):1709–1715 22863869 10.1016/j.urolonc.2012.06.014

[CR15] Pritchard EM, Dyer MA, Guy RK (2016) Progress in small molecule therapeutics for the treatment of retinoblastoma. Mini Rev Med Chem 16(6):430–454 26202204 10.2174/1389557515666150722100610 PMC5509337

[CR16] Romani P, Valcarcel-Jimenez L, Frezza C, Dupont S (2021) Crosstalk between mechanotransduction and metabolism. Nat Rev Mol Cell Biol 22(1):22–38 10.1038/s41580-020-00306-w 33188273

[CR17] Roy SR, and Kaliki S (2021) Retinoblastoma: a major review. Mymensingh Med J 30(3):881–895 34226484

[CR18] Russo E, Spallarossa A, Tasso B et al (2022) Nanotechnology for pediatric retinoblastoma therapy. Pharmaceuticals 15(9):1087 36145308 10.3390/ph15091087 PMC9504930

[CR19] Sharma A, Alswillah T, Singh K, Chatterjee P, Willard B, Venere M, Summers MK, Almasan A (2018) USP14 regulates DNA damage repair by targeting RNF168-dependent ubiquitination. Autophagy 14(11):1976–1990 29995557 10.1080/15548627.2018.1496877 PMC6152509

[CR20] Shields CL, Lally SE, Leahey AM, Jabbour PM, Caywood EH, Schwendeman R, Shields JA (2014) Targeted retinoblastoma management: when to use intravenous, intra-arterial, periocular, and intravitreal chemotherapy. Curr Opin Ophthalmol 25(5):374–385 25014750 10.1097/ICU.0000000000000091

[CR21] Wall NR, O’Connor DS, Plescia J, Pommier Y, Altieri DC (2003) Suppression of survivin phosphorylation on Thr34 by flavopiridol enhances tumor cell apoptosis. Cancer Res 63(1):230–235 12517802

[CR22] Wu X, Qin L, Fako V, Zhang JT (2014) Molecular mechanisms of fatty acid synthase (FASN)-mediated resistance to anti-cancer treatments. Adv Biol Regul 54:214–221 24080588 10.1016/j.jbior.2013.09.004

[CR23] Yang WS, SriRamaratnam R, Welsch ME, Shimada K, Skouta R, Viswanathan VS, Cheah JH, Clemons PA, Shamji AF, Clish CB, Brown LM, Girotti AW, Cornish VW, Schreiber SL, Stockwell BR (2014) Regulation of ferroptotic cancer cell death by GPX4. Cell 156(1–2):317–331 24439385 10.1016/j.cell.2013.12.010 PMC4076414

[CR24] Yang Jiaojiao JA (2017) Research progress of Survivin in malignant tumors. Inner Mongolia Med J 49(6):679–682

[CR25] Zhu Y, Zhang C, Gu C, Li Q, Wu N (2016) Function of deubiquitinating enzyme USP14 as oncogene in different types of cancer. Cell Physiol Biochem 38(3):993–1002 26938858 10.1159/000443051

